# Bidirectional control of a one-dimensional robotic actuator by operant conditioning of a single unit in rat motor cortex

**DOI:** 10.3389/fnins.2014.00206

**Published:** 2014-07-25

**Authors:** Pierre-Jean Arduin, Yves Frégnac, Daniel E. Shulz, Valérie Ego-Stengel

**Affiliations:** Unité de Neuroscience, Information et Complexité, Centre National de la Recherche ScientifiqueGif-sur-Yvette, France

**Keywords:** brain-machine interface, neuroprosthesis, learning, neuronal plasticity

## Abstract

The design of efficient neuroprosthetic devices has become a major challenge for the long-term goal of restoring autonomy to motor-impaired patients. One approach for brain control of actuators consists in decoding the activity pattern obtained by simultaneously recording large neuronal ensembles in order to predict in real-time the subject's intention, and move the prosthesis accordingly. An alternative way is to assign the output of one or a few neurons by operant conditioning to control the prosthesis with rules defined by the experimenter, and rely on the functional adaptation of these neurons during learning to reach the desired behavioral outcome. Here, several motor cortex neurons were recorded simultaneously in head-fixed awake rats and were conditioned, one at a time, to modulate their firing rate up and down in order to control the speed and direction of a one-dimensional actuator carrying a water bottle. The goal was to maintain the bottle in front of the rat's mouth, allowing it to drink. After learning, all conditioned neurons modulated their firing rate, effectively controlling the bottle position so that the drinking time was increased relative to chance. The mean firing rate averaged over all bottle trajectories depended non-linearly on position, so that the mouth position operated as an attractor. Some modifications of mean firing rate were observed in the surrounding neurons, but to a lesser extent. Notably, the conditioned neuron reacted faster and led to a better control than surrounding neurons, as calculated by using the activity of those neurons to generate simulated bottle trajectories. Our study demonstrates the feasibility, even in the rodent, of using a motor cortex neuron to control a prosthesis in real-time bidirectionally. The learning process includes modifications of the activity of neighboring cortical neurons, while the conditioned neuron selectively leads the activity patterns associated with the prosthesis control.

## Introduction

Neuronal operant conditioning consists in training a freely behaving animal so that the firing rate of a preselected neuron, recorded simultaneously to the behavior, modulates to achieve goals set by the experimenter. Provided that the human or animal is properly rewarded, it has been shown that the activity of neurons could be conditioned in different zones of the brain (Olds, 1965; Shinkman et al., 1974; Marzullo et al., 2006; Cerf et al., 2010; Kobayashi et al., 2010; Schafer and Moore, 2011; Sakurai and Takahashi, 2013), including the motor cortex (Fetz, 1969), and with high success and short training time in the monkey (Moritz et al., 2008; Moritz and Fetz, 2011). This plastic capability of the neuron to adapt to a new task, and display relatively fast and precise modulations has been proposed to be usable to drive real-time prosthetic devices for a long time (Schmidt, 1980). However, it was only in 2008 that operant conditioning of one neuron served as a way to control, by electrical stimulation proportional to the cell's activity, the contraction of the wrist muscles of a monkey (Moritz et al., 2008). More recent studies have shown that abstract skills, such as the control of an auditory pitch, could also be learnt through operant conditioning of one or a few neurons (Gage et al., 2005; Koralek et al., 2012).

During the last decade, a parallel approach has been developed, based on the decoding of the activity of a larger population of neurons to reconstruct a limb movement (Chapin et al., 1999). Brain-machine interfaces of increasing complexity have been implemented (Carmena et al., 2003; Velliste et al., 2008; Pohlmeyer et al., 2009; Suminski et al., 2010), that already prove useful for human subjects (Hochberg et al., 2012; Collinger et al., 2013). In practice, the decoding strategy does not always allow control of the device immediately and may necessitate a period of several days or weeks of training (Taylor et al., 2002; Ganguly et al., 2011; but see Serruya et al., 2002, for rapid control of a 2D-cursor). This is especially true for motor-impaired human subjects, for which the optimization of the brain control algorithm cannot rely on daily determination of functional properties of the recorded neurons. Increasing the number of dimensions of the prosthesis, as has been achieved in a recent human study using a prosthetic arm with seven degrees of freedom (Collinger et al., 2013), requires increasing the amount of training in order to master the robotic device. Also, at present, a robotic arm can perform a food-grasping task with success but without the smoothness and the speed of a real arm (Velliste et al., 2008), although promising progress has been achieved with sophisticated algorithms for control of a 2D cursor (Gilja et al., 2012). We propose that strategies to control simple prosthetic devices (in 1D) with time-scales compatible with muscle-control reactivity and stability should first be designed, before increasing the number of degrees of freedom. To that end, the performance of one or a small group of neurons, trained to drive a low dimension device through operant conditioning, should be assessed quantitatively.

Neuroprosthetic training protocols likely all trigger various forms of plastic modifications distributed in the brain. These functional changes, particularly of the neurons whose activity directly drives the prosthesis, contribute to the successful control of the brain machine interface (Taylor et al., 2002; Ganguly and Carmena, 2009). In neuronal operant conditioning, it has been reported early on that cells immediately adjacent to a conditioned unit, but not included in the reinforcement contingency, tend to exhibit correlated activity (Fetz and Baker, 1973). More recently, several studies have examined in more detail the extent to which surrounding neurons in the network also modify their functional properties. In two different experiments in the monkey motor cortex, Carmena and collaborators showed that neurons not directly relevant for the prosthesis control exhibited marked changes in activity during the brain control trials (Ganguly et al., 2011; Koralek et al., 2013). However, neurons directly controlling the prosthesis exhibited a higher modulation of activity (Ganguly et al., 2011), and a precise temporal coherence with downstream pathways absent in neighboring neurons (Koralek et al., 2013). In a previous study, we have also reported the presence of changes in the spiking activity of neurons not directly controlling a prosthetic device, but to a lesser extent than the neurons directly relevant to the behavioral output (Arduin et al., 2013). Andersen and collaborators have argued that the activity patterns taking place during brain control of neuroprostheses belong to the natural movement repertoire of the motor pathways (Hwang et al., 2013). We believe that concomitant changes in the motor network need to be assessed more systematically, particularly in order to evaluate whether it will be feasible to recruit independently different populations of neurons in the same area for the different dimensions of control of a single prosthetic device.

In order to address these questions, we designed a neuronal operant conditioning protocol using rats, employing only one motor cortex neuron at a time. In our previous study (Arduin et al., 2013), the discharge of the conditioned neuron was controlling a one-dimensional actuator on which a bottle containing a liquid reward could move in one direction. We showed that after learning, the neuron indeed raised its activity during the trials. This increase of discharge occurred as a transient burst shortly after trial onset. It remained to be determined whether the neuron's spiking activity could be maintained for a prolonged period, and thus achieve a stable and continuous control of the bottle position over time. In this study, we present results of a bidirectional version of the operant conditioning task in which the bottle could move in both directions depending on whether the firing rate of the conditioned neuron increased or decreased. Thus, the neuronal firing rate had to adapt in real-time in order to stabilize the bottle position in the drinking zone.

## Materials and methods

### Animal handling and pre-training

**Figure 1:**
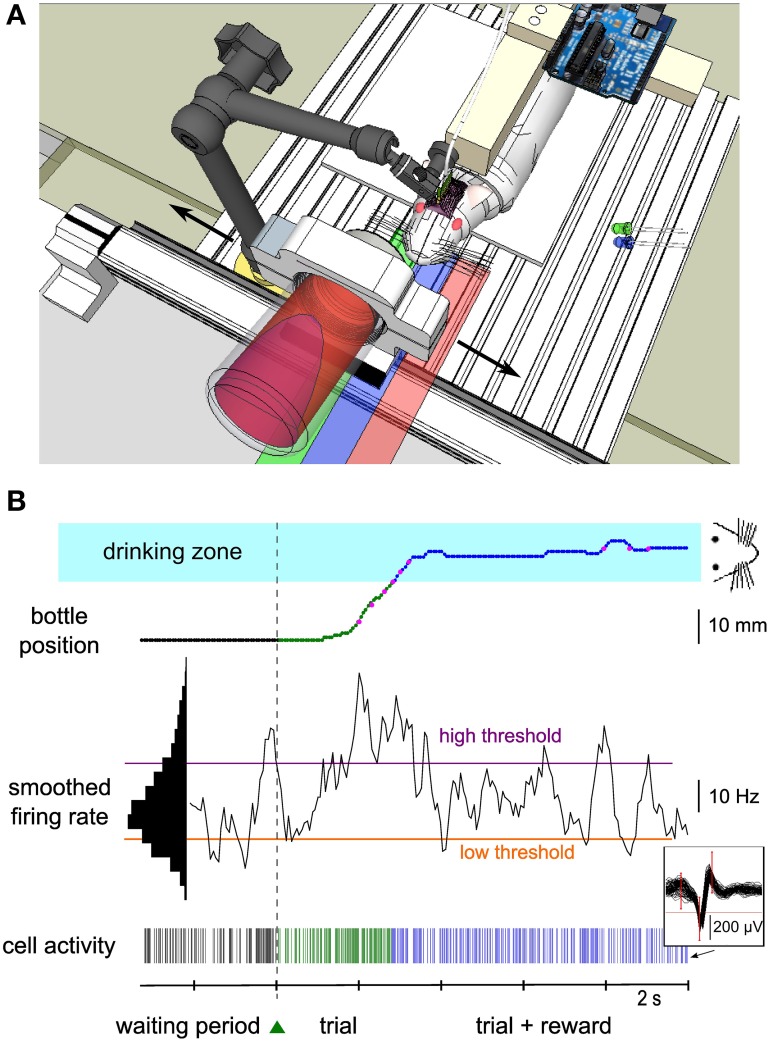
**Experimental setup of the neuronal control protocol. (A)** Schematics of the experimental setup. The rat was suspended in a harness attached on a PVC bar. Its hindlimbs touched a platform while its forelimbs were free to move. The head was fixed by a mechanical arm that was plugged to a PVC piece embedded into the dental cement of the implant. A bottle containing water and syrup was held by a metal piece placed on a one-dimensional linear axis, perpendicular to the rat's body. A green LED was placed on the left of the animal. It was switched on and off by a microcontroller mounted on a printed circuit board near the animal to indicate start and end of each trial. The bottle could move in the two directions (black arrows) and the rat could drink when the bottle was close enough to the center (“drinking zone,” blue shaded area). The green and red shaded areas represent the right and left zones explored by the bottle. **(B)** Bottom, spiking activity of a single neuron (see 60 superimposed action potentials in the inset) during the waiting period (black ticks), during the trial before reward is reached (green ticks) and during the rewarded part of the trial (blue ticks). The smoothed firing rate of the unit (middle) controlled the speed of the bottle toward the rat from a lateral starting position (top). The color of the bottle position curve matches the color of the spiking activity at that time and indicates the waiting/trial status and the bottle position zone in the trial. Magenta dots indicate key positions recorded during the experiment, while the rest of the curve was reconstructed offline (see Methods). The speed of the bottle depended on the difference between the firing rate and two thresholds (purple and orange horizontal lines). Those thresholds were set at fixed percentiles of the firing rate distribution (left black histogram, see Methods). When the bottle was in the drinking zone (blue shaded area), the rat was able to drink while still controlling the bottle position. The green triangle between the waiting period and the trial represents the LED that was switched on to indicate trial onset. Color conventions for the position zones and activity thresholds apply to all figures.

Three male Wistar rats weighing 250–350 g were obtained from our in-house animal facility (French Agriculture Ministry Authorization C91-272-105). Maintenance, manipulations and surgery were performed in conformity with French (JO 2001-464) and European legislation (2010/63/UE) on animal experimentation. Before surgery, animals were progressively trained to stay quietly in a harness and drink from a bottle containing a solution of water and glucose (strawberry syrup). While attached in the harness, the posterior limbs laid on a platform, and the forelimbs were free to move. Animals were kept at 85% of their free-feeding weight. The bottle was mounted on a one-dimensional linear actuator (Festo, Germany) perpendicular to the rat body, and moved to and away from the rat mouth on a left-right lateral axis (Figure 1A). During the pre-operative training phase (pre-training), the bottle followed four successive steps per trial: (1) a waiting period of 8–12 s in the dark, during which the bottle was kept away from the animal; (2) a fast displacement of the bottle to the mouth position during which a green light-emitting diode (LED) placed close to the animal was “on”; (3) a period of 3 s of drinking during which a blue LED was “on”; (4) a return travel, back to the initial start position. A new waiting period then started. Two sessions of 10–15 min occurred each day, consisting of ~50 trials each. The LEDs were switched “on” and “off” by a microcontroller (Arduino Diecimila, Italy). The whole pre-training period lasted several weeks.

### Surgical procedure

Two days prior to surgery, the rat received subcutaneous injections of 0.1 mL of the anti-inflammatory drug meloxicam (Metacam 0.5 mg.kg^−1^) and 0.1 mL of the antibiotics drug cevofecin (Convenia 25 mg.kg^−1^) to prevent pain and infections, respectively. We placed the animal in a ventilated box and induced anesthesia with isoflurane at 3%. The animal was then transferred to a stereotaxic frame. The ear bars were covered with lidocaine gel (Xylocaine). Anesthesia was maintained throughout surgery with isoflurane. The level of isoflurane was progressively decreased down to ~1.5%. We injected 0.3 mL of lidocaine 2% under the head skin before incision. Once the skull was exposed, seven to eight screws were inserted, both to ensure a strong contact between skull and implant, and for electrical grounding (see below). A craniotomy was drilled above the limb region of the motor cortex (A1.5, L3.0), and the dura was resected. The rats were implanted with microwire arrays of 8 rows and 4 columns with a grid spacing of 0.25 mm (Microprobes for Life Sciences, MD, USA). The electrodes were lowered to a cortical depth of ~1300 microns. Once the microwire array was in place, a ground wire was coiled around one or several ground screws (Phymep, France). Gelfoam was applied around the upper part of the electrodes outside of the brain to help prevent bleeding. Drops of cyanoacrylate were sparsely spread on the dry skull. The remaining skull area was then covered with dental acrylic (Henry Schein, USA) together with the base of the electrode implant. Finally, a piece of polyvinyl chloride (PVC, custom-made) was embedded in the dental cement to allow head-fixation in subsequent training sessions (see below). The rat received a saline injection intraperitoneally before the anesthesia was stopped. Food was accessible *ad libitum* for 5 days during which the rat was closely looked after to check proper recovery. Drops of an oral solution of meloxicam were given if signs of pain or disturbance were noticed.

### Head fixation

The rat was then submitted to the same food deprivation protocol as before surgery. The training sessions were similar and the rat was taught to accept a strict head-fixation ensured with a 3D articulated arm (NMG700030, Noga, Germany) whose extremity (NFA1100) mated with the PVC piece glued to the skull (Figure 1A). With that device, the rat's mouth could be positioned precisely by the experimenter in front of the bottle in the drinking position. The four limbs were still free to move as above.

### Data acquisition and control of the behavioral setup during training

Neuronal activity was recorded and processed in real-time (Cerebus hardware, Blackrock Microsystems, UT, USA). Each electrode output was filtered between 250 Hz and 7.5 kHz, and sampled at 30 kHz. Spikes were sorted online (Central software, Blackrock Microsystems). Spike sorting was performed at the beginning of each session, using a template-matching method: assignment of a waveform to a unit depended on whether it crossed all the criterion windows drawn by the experimenter (Figure 1B, inset). A putative unit was considered as well isolated if less than one percent of spikes was contained in the first bin (2 ms) of its autocorrelogram. For one conditioned unit, we could not reliably separate the spikes corresponding to two different waveforms; therefore we classified this unit as a multi-unit. Spikes were considered to be emitted by the same unit from one session to the next when their waveform remained invariant (Supplementary Material). This was evaluated by checking that the average waveforms, normalized by their peak value, were superimposed within ~10% of each other. A change in peak amplitude was allowed as long as the spikes could be well isolated. Spikes of non-conditioned neurons were not always successfully isolated throughout all successive sessions for the currently conditioned neuron. All information was sent to a computer (Dell Intel QuadCore at 2.66 GHz, 3.24 Gb of RAM, OS Windows XP) via a fiber-optic data link. A custom-made software (Eclipse Qt C++) read in the spike information in real-time and commanded the linear actuator holding the bottle through a serial 56 k baud communication. For technical reasons, we could not record the bottle position continuously during the session. Instead, the instantaneous bottle position was recorded on the neural data file through a second serial port whenever the bottle crossed from one spatial bin to another (13 bins spanning the bottle course).

### Neuronal control of the bottle position

A single unit was chosen as the operantly-conditioned neuron for controlling the bottle position. Criteria for selection were stability of recording over days, high signal-to-noise ratio, wide firing rate distribution and modulation with forelimb movement. During the experiment, spiking activity was computed every 62.5 ms, and was smoothed over 500 ms by convolving each spike with a continuous filter: h(t) = 2 * (0.5 − t) if t was between 0 and 0.5 s, and h(t) = 0 otherwise. Neuronal control consisted in using this smoothed firing rate to determine the bottle speed and direction.

The full training of one neuron was composed of three successive phases, each consisting of many sessions spread over several days or weeks. In the first training phase, the automatic displacement of the bottle present during pretraining before surgery was now replaced by neuronal control. The firing rate of the conditioned neuron needed to increase above a “high” threshold chosen by the experimenter (see below) to make the bottle move toward the rat's head, and allow eventually liquid reinforcement once it was in a specific zone close enough to the mouth. This “drinking zone,” indicated by a blue rectangle on all figures, extended over 8 mm on each side of the mouth (total length = 16 mm), which represented 34% of the total bottle course. It could be slightly off-centered, depending on the exact head-fixation configuration. Once the neuron was significantly conditioned in this unidirectional movement task, we moved on to the next phase of training in which we introduced a punishment rule: if the smoothed neuronal firing rate was below a “low” threshold, also defined by the experimenter, the bottle moved now in the opposite direction, that is, back toward its initial position and away from the mouth. In addition, the bottle speed “v” depended linearly on the smoothed activity “f” when above or below the two thresholds f_low_ and f_high_ according to the following relation (see **Figure 4A** for a graphical display of this function):

$$\begin{array}{l} \text{v}(\text{f})=-{\text{v}}_{0}.(\text{f}-{\text{f}}_{\text{low}})/({\text{f}}_{\text{high}}-{\text{f}}_{\text{low}})\text{ if f}\geq {\text{f}}_{\text{high}},\\ \text{v}(\text{f})={\text{v}}_{0}.({\text{f}}_{\text{high}}-\text{f})/({\text{f}}_{\text{high}}-{\text{f}}_{\text{low}})\text{ if f}\leq {\text{f}}_{\text{low}},\\ \text{v}(\text{f})=0\text{ otherwise}, \end{array}$$

where v_0_ is a speed scaling factor that was progressively decreased, from ~3 to ~1 cm.s^−1^ as training progressed. The low and high thresholds were re-evaluated every block of 3 successive trials. Their value was set respectively to 10 and 90% of the firing distribution during a time period spanning approximately the previous 15 trials (including waiting and trial periods). Whenever the control algorithm returned a position outside of the defined boundaries of the rail, the speed was automatically set to 0. If the bottle arrived in the drinking zone within the trial duration, then the rat was allowed to drink for 3 s (step 3, similarly to pre-surgery).

Once this second phase of training had been completed, the full bidirectional control of the actuator could start. All the data described in the Results are from this last phase of training, realized on seven units. These units belong to a larger data set of 17 neurons recorded from eight rats that were trained to drive an actuator unidirectionally (Arduin et al., 2013). The neuron activity during each trial controlled the speed of the bottle in both directions according to the control algorithm given above. However, unlike phase 2, the trial did not stop anymore when the bottle reached the drinking zone: instead, the bottle position had to be maintained by neuronal control in that region of space, allowing the rat to drink (Figure 1B). After the trial (duration 10 s), the bottle automatically went back to its starting position. Because of the difficulty of the task, an automatic help was introduced, by adding a bias to the speed of the bottle in the first sessions: part of the speed vector was determined by the neuronal control algorithm, and part of it, the bias term, pointed toward the drinking zone, with a relative strength proportional to the distance between the bottle and the mouth. This bias was progressively decreased to zero before the full bidirectional sessions could be recorded. The high and low thresholds were still re-evaluated periodically as in the second phase, by being drawn from the binned distribution of firing rate established from approximately the 15 previous waiting periods. Because of this long integration period and the binning of firing rate, thresholds were in fact modified on average only every 19 trials, and the mean threshold change was 11% (as calculated on the seven best sessions, as defined below).

The number of sessions during which we collected data was 2, 18, 9, 7, 34, 10, and 8 for the seven neurons (mean = 12.6 sessions per neuron). The total training time of a neuron, from animal handling to the first bidirectional session, could last up to 3 months.

### Reconstructed bottle trajectories

All spiking activities were analyzed with a custom-made program (Eclipse Qt C++). Results were displayed with the same software or in Matlab (Mathworks Inc., MA, USA). For each session, we calculated the total time within trials during which the bottle was positioned in the drinking zone. In order to compare this time to a control situation, we calculated virtual bottle trajectories and corresponding drinking time using the neuronal activity of a control dataset (see below for the two control datasets used). Prior to assessing significance between control and test data, we needed to verify the validity of the offline algorithm of trajectory calculation. Thus, we tested it by reconstructing bottle positions using the spiking activity of the conditioned neuron recorded during trials, and comparing them to the real ones. Indeed, the two simulated and measured trajectories matched, as can be seen by comparing the magenta dots of the real trajectory to the reconstructed blue curve on Figure 1B. Occasionally, we observed small differences due to delays of one clock increment (62.5 ms) in the online control algorithm (Arduin et al., 2013). Comparison of the corresponding “reconstructed” drinking time with the real time spent in the drinking zone confirmed nonetheless the accuracy of our procedure (relative error: 1.6 ± 0.3%). Two control sets of bottle trajectories were then computed. For the first set, we used the activity of the conditioned neuron recorded during the waiting periods of the same session. For the second set, we reconstructed the bottle positions with the trial activity of the conditioned neuron after shuffling it by blocks of 2 s within each trial. That way, we kept the general statistics of the spike train of the neuron, in particular its mean firing rate during trials, and assessed whether temporal changes in activity were generating the successful control of the bottle. This bootstrap test was conducted 10,000 times for each session. For analyses requiring the best session per neuron, it was defined as the session for which the real drinking time differed the most from this bootstrap distribution of reconstructed drinking times.

### Neuronal activity vs. bottle position

We calculated the statistics of the firing rate as a function of the bottle position. Values were grouped in 13 position bins of increasing size when moving away from the rat's mouth. To assess experimental biases, we used again the control dataset composed of the activity of the conditioned neuron recorded during the waiting periods of the same session, and the corresponding reconstructed bottle positions. However, those control trajectories were often confined very close to the starting position, so that the full control curve of average neuronal activity vs. position could not be estimated accurately. In order to explore the full course of the bottle, we shuffled the waiting period activity by blocks of 2 s. One thousand shuffle datasets were sufficient to obtain the control curve. We observed discontinuities in the activity curves for the bins at the boundaries of the position range, whether with the real trial activity or a control activity. This was due to the fact that the algorithm did not allow the bottle to move out of the preset boundaries. For example, if the firing rate persisted under the low threshold while at the extreme right position, the bottle could not move toward the right and low activity values accumulated for that bin until the firing rate rose again. For regression analysis of activity vs. position, we removed the boundary bins showing a discontinuity in the control curve (e.g., **Figure 4D**, right side only). The criterion for determining the presence of a discontinuity was that the gap between the boundary bin and the next one was more than 2 times the maximum gap of the non-boundary bins. Regression analysis was performed separately for position values on the two sides of the rat's mouth.

### Mean latency across session and rank of activation

To compute the response latency for a given session and a given neuron (conditioned or not), a peri-event time histogram (PETH) between −2 s and +2 s was constructed, centered on trial start. We used sliding windows of 100 ms, with 20 ms steps. The mean of the PETH was computed within [−2 0 s] to assess the baseline average firing rate before trial onset (mean ± SD). We compared the activity in each bin after trial onset with the activity before trial. The latency was calculated in two steps, by finding: first, six successive PETH z-scores greater than 2; second, within this window of six bins, with a 20 ms bin (non-sliding) PETH, the first z-score greater than 1.

The session latency was computed for all recorded neurons exhibiting an increase of activity after trial onset above 2SD. A previous study (Arduin et al., 2013) demonstrated that trial-to-trial variability before trial start can be very high, so that clear increases in PETHs did not always reach high z-score values. Nonetheless, in order to detect response latencies for neurons with high variability, we used for each neuron the variance it would have had if it fired like a Poisson process. If during a session, a latency could be defined for at least one neuron, we looked at the order of activation of all other neurons simultaneously recorded at that session based on their session latency. If several neurons had the same latency, their rank of activation was defined as the average of the ranks of those neurons (rounded to the nearest integer).

## Results

### Neurons control in real-time a one-dimensional actuator

**Figure 2:**
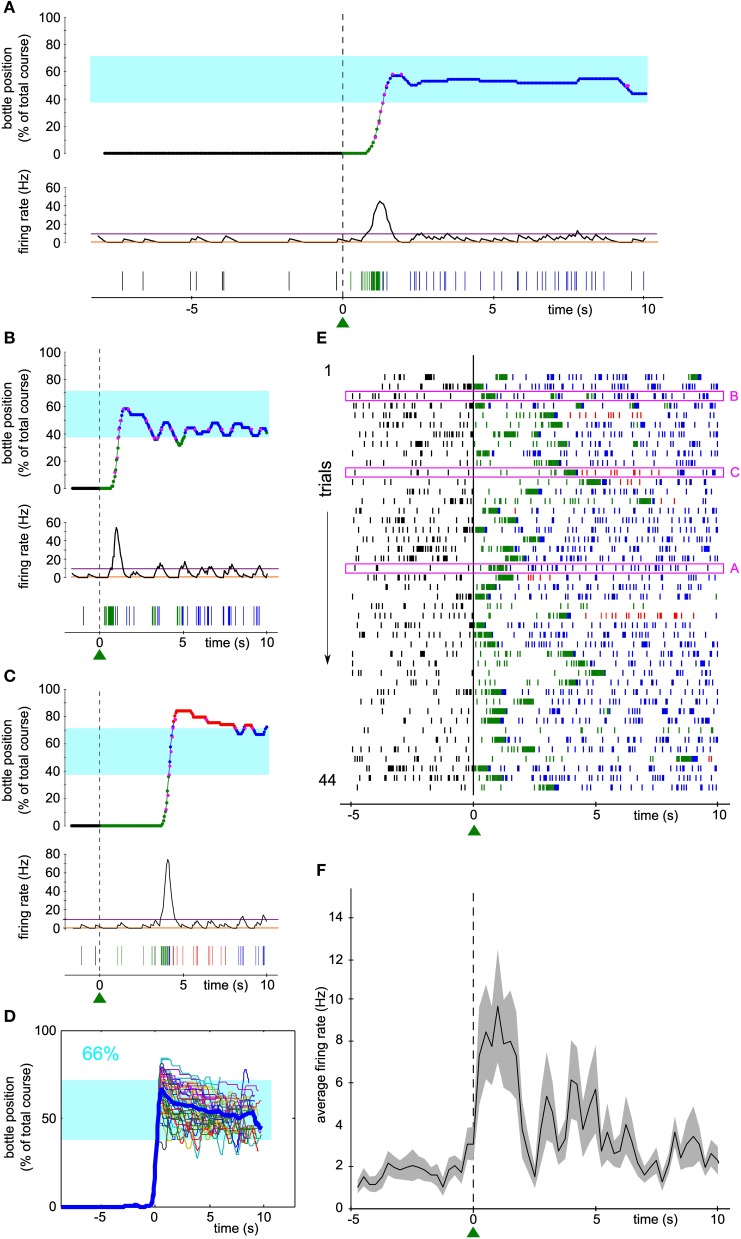
**Activity of a single unit controls the bottle position in real time to allow drinking. (A–C)** Top, reconstruction of the bottle movement during three trials for one neuron during one session, color coded according to waiting/trial status and bottle position zones as in Figure 1. Middle, smoothed firing rate and thresholds used by the neuronal control algorithm. Bottom, spikes recorded during the corresponding waiting periods and trials. The activity-based reconstructed position is compared to the real position of the bottle recorded during the time course of the trial at some key ordinates (magenta dots). **(D)** Superposition of all reconstructed trajectories for that session, centered in time on the position *y* = 20 % for clarity, if the bottle reached that position (95% of the trials matched that condition; *n* = 42/44). The percentage of total trial time spent in the drinking zone is displayed in the upper left corner in blue. In this and the following Figures, the thick blue line is the average trajectory. **(E)** Raster plot of the activity of the conditioned neuron around trial start (*t* = 0). Ticks represent spike times and are colored depending on the bottle position as before. The three trials highlighted in magenta rectangles are the ones depicted in **(A–C)**. **(F)** Peri-event time histogram (PETH) of the conditioned neuron activity averaged across all trials of the raster plot shown in **(E)** (bin size: 200 ms). The shaded area indicates ± s.e.m.

One hundred and fifty-five neurons have been recorded from three rats. Seven units (6 single-units, 1 multi-unit) were selected, one at a time, to control a one-dimensional actuator in real-time. In the bidirectional task described herein, the selected neuron was required to modulate its firing rate during trials of 10 s in order for the rat to get liquid reinforcement. The reward bottle was mounted on a rail and could either move left, right, or stay still depending on the level of the neuronal activity compared to a low and a high threshold (see Methods and Figure 1). Figures 2A–C show three examples of the bottle trajectories controlled by the brain-machine interface and corresponding to three trials during one session. The spiking activity and smoothed firing rate of the conditioned neuron are displayed below the corresponding trajectories. On the first trial plotted (Figure 2A, top), the bottle trajectory quickly reached the drinking zone, and stayed there for the rest of the trial. The corresponding activity showed a sharp burst (Figure 2A, middle and bottom) after trial onset, and then a stable firing rate in between thresholds, higher than during the waiting period. This indicates that the neuron's firing rate adapted in real-time to the position of the bottle in order to maximize the drinking time. In another trial, a first burst of activity brought the bottle in the drinking zone, and was followed by smaller bursts which allowed the bottle to stay in the drinking zone for almost 10 s (Figure 2B). Still in other trials, the initial burst of spikes led to overshooting so that the bottle quickly crossed the drinking zone to the other side. It was followed by low activity so that eventually the bottle converged back to the drinking zone (Figure 2C). An overlay of all trajectories for that session, aligned on their crossing above 20% of the full bottle course, shows that the bottle consistently moved to the drinking zone, and that afterwards it mostly stayed inside that zone for the rest of the trials (Figure 2D). Small deviations outside of the drinking zone were quickly corrected on both sides. Overall, the bottle was inside the drinking zone for 66% of the total trial time for that session.

Figure 2E shows a raster plot of the activity of the conditioned neuron for that session, centered on trial start. Large modulations of activity can be noticed, each time allowing the bottle to enter the drinking zone (transition from green to blue spikes in the raster), with a variable latency after trial onset. The firing rate then stabilized and the rat could drink from the bottle, as indicated by the blue spikes. Plotting the average firing rate across trials confirmed that the large bursts of activity occurred mainly in the first few seconds after trial start, followed by a period of variable spiking activity at an intermediate firing rate level (Figure 2F).

In order to estimate the performance of the conditioned neuron, we designed control datasets with which to compare the trial trajectories. We simulated the movements of the bottle that would have been produced by the neural control algorithm (1) had we taken the activity of the conditioned neuron during the waiting period as input, and (2) with the trial activity shuffled by blocks of 2 s as input.

**Figure 3:**
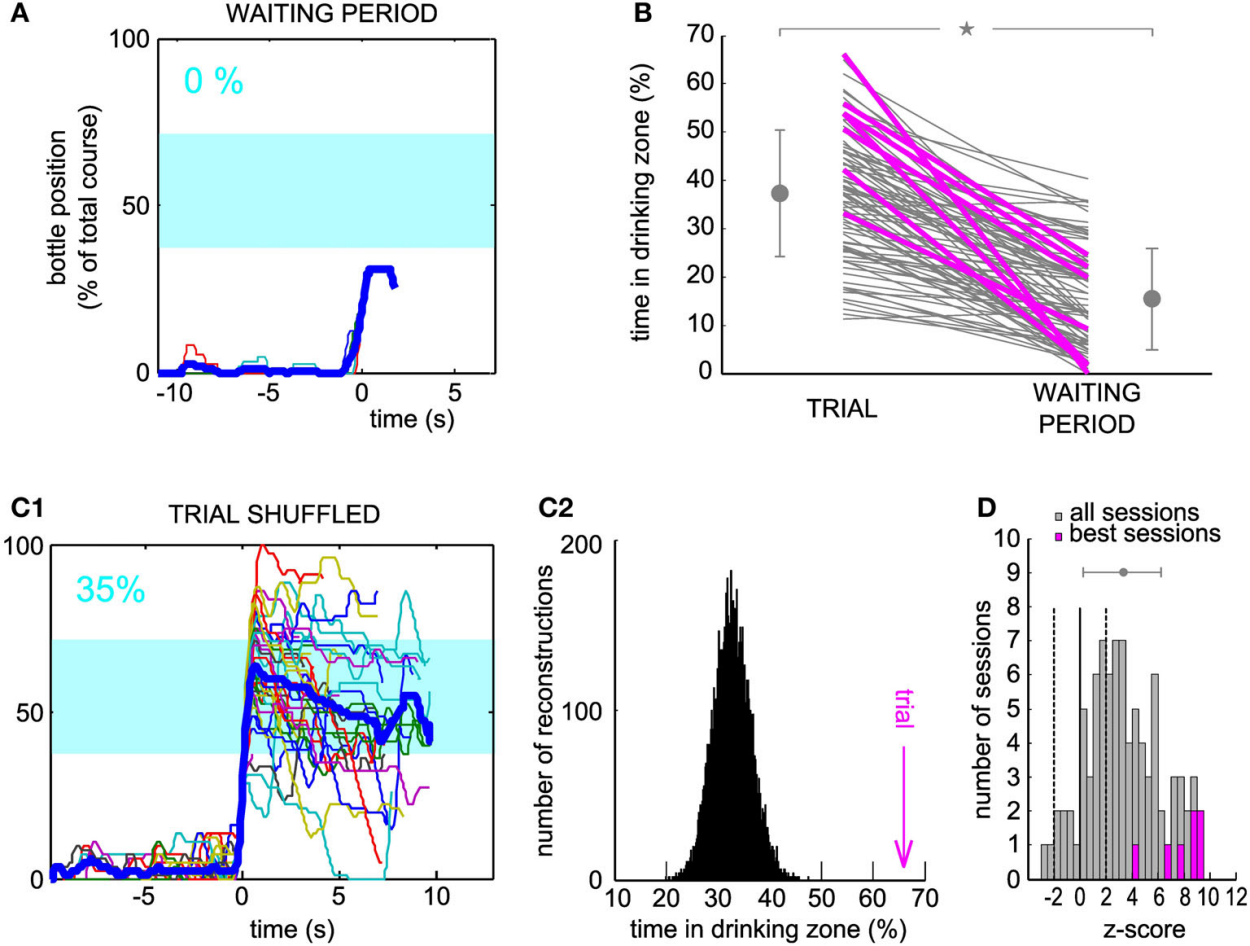
**Neuronal control of the bottle resulted in a higher task performance than in control datasets. (A)** Superposition of all reconstructed trajectories for the same conditioned neuron and the same session as Figure 2D, centered in time on the position *y* = 20% for clarity, if the bottle reached that position (11% of the trials only, the rest was not plotted), using the waiting period activity as an input to the neuronal control algorithm. The percentage of total trial time spent in the drinking zone is displayed in the upper left corner in blue. **(B)** Percentage of time spent in the drinking zone for the trial datasets and the waiting period (control) datasets. Each line joins the two values calculated for a conditioned neuron during one session. Magenta lines are the best sessions (one per neuron). The mean ± SD are indicated on each side of the graph for Trial and Waiting (gray dots and error bars). The star indicates a significant difference (paired sample Wilcoxon test, *P* < 5.10^−16^). **(C1)** Same as **(A)**, but the activity used for trajectory reconstruction was the trial activity, shuffled by blocks of two seconds (93% of trials plotted). **(C2)** Distribution of the drinking time percentage obtained in **(C1)**, with a bootstrap analysis of 10,000 shuffles. The value found with real trial activity is indicated by the vertical magenta arrow and corresponds to a z-score of 9.2. **(D)** Distribution of the z-scores of the real time spent in the drinking zone compared to the bootstrap distribution for shuffled activity, for all the sessions. Magenta, histogram for the seven best sessions (one per neuron). Dashed lines, z-score = −2 and 2; full line, z-score = 0. The gray dot and error bar indicate the mean ± SD.

For the neuron and the session displayed in Figure 2, the arrival into the drinking zone would never have been possible had the bottle speed been controlled by the activity of the waiting period (Figure 3A). We found that for a large majority of the sessions (86/88), the performance was higher using real trial activity than using waiting activity, that is, the trajectories simulated with the waiting period spiking activity stayed less time in the drinking zone (Figure 3B, only two lines have a positive slope, 86 have a negative slope). A paired comparison of the two groups of values confirmed that this effect was significant across all sessions (paired sample Wilcoxon test, *P* < 5.10^−16^). The magenta lines indicate one session per neuron (the “best” session, as determined with the second control dataset, see Methods and below).

One possible caveat could have been that overall changes in firing rate between waiting period and trial might be responsible for this difference in performance. Thus, we looked at the trajectories generated from the trial activity after shuffling it in time (see Methods), for which, by construction, the average firing rate was the same as during the real trials. For the shuffle dataset displayed on Figure 3C1, and corresponding to the same neuron and session as Figures 2, 3A, almost all trajectories entered the drinking zone at some point in time, but they tended to diverge randomly afterwards and did not stay inside as long as the real trial trajectories (compare with Figure 2D). The control drinking time that resulted was thus smaller than the real drinking time (35 vs. 66% of total trial time). We quantified the performance of the neuron by comparing the drinking time using trial activity to the distribution of drinking times obtained when repeating the shuffled procedure 10,000 times and found a highly significant z-score value, above 9, for this neuron and session (Figure 3C2). This analysis was applied on the 88 sessions. The z-score was above 2 for 58 sessions (Figure 3D), and averaged to 7.8 on the seven best sessions (one per neuron, defined as the session for which the z-score was maximal). Across all sessions, the mean of the distribution of z-scores was significantly higher than 0 (Student's *t*-test, *P* < 7.10^−18^). However, there was no significant increase in the z-score between the first session (mean ± SD, 2.5 ± 3.7) and the seventh session (mean ± SD, 3.8 ± 2.2, Wilcoxon paired test, *P* = 0.32, *n* = 6 neurons tested for 7 sessions or more). This could be due to the small size of our sample of neurons, probably insufficient to reveal a gradual improvement over sessions. We conclude from these controls that the temporal patterns of neuronal activity produced during trials effectively controlled the bottle position, in a way that optimized the time spent in the drinking zone.

### Encoding of the bottle position by the neuronal firing rate

**Figure 4:**
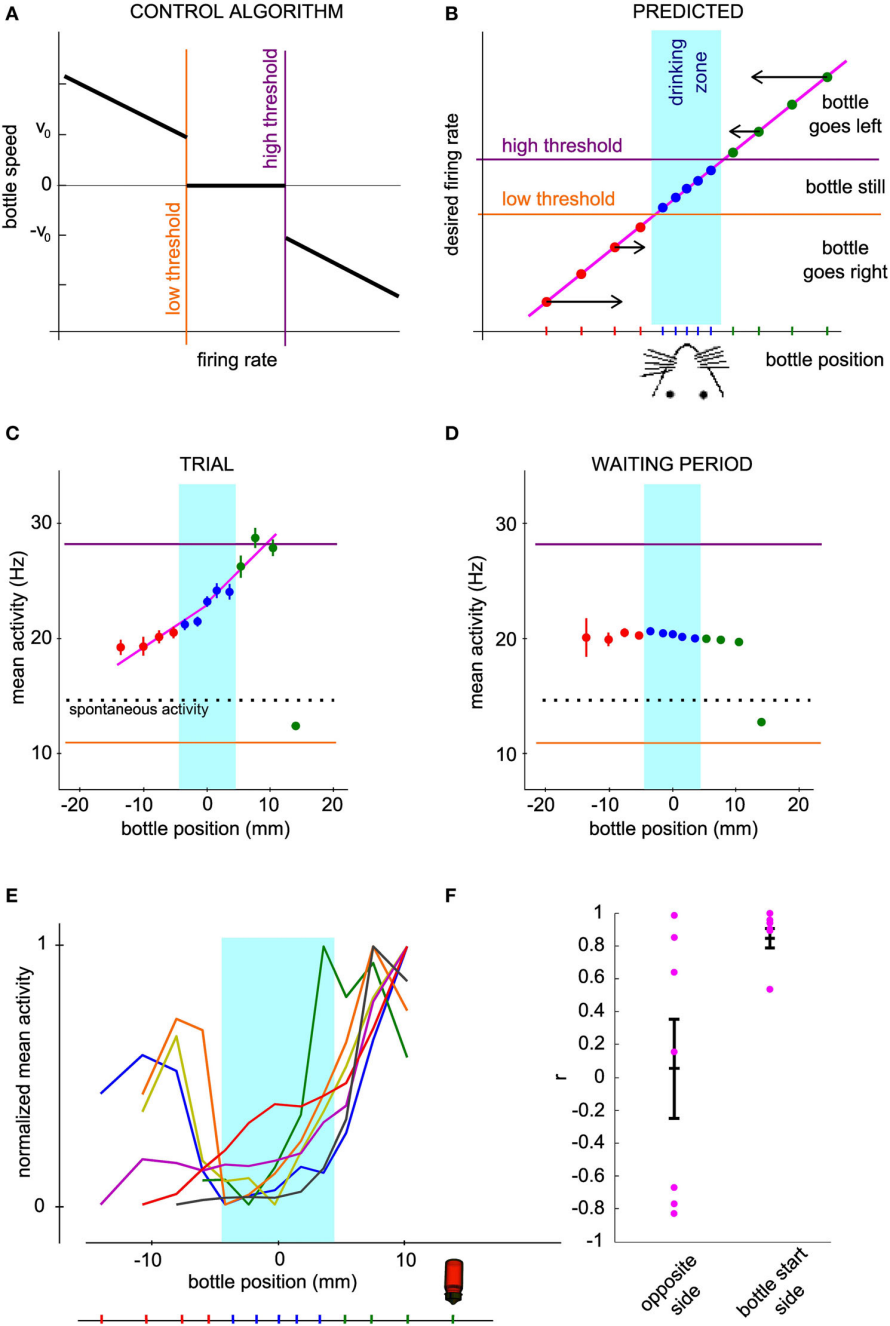
**The mean neuronal activity encodes bottle position. (A)** Control algorithm giving the bottle speed and direction of movement as a function of the neuronal activity relative to the low and high thresholds. **(B)** Predicted relation between neuronal activity and bottle position for successful operant conditioning. The arrows qualitatively show the optimal direction and speed of the bottle for each bottle position, converging to the rat mouth. The vertical location of the points is thus determined by the firing rate required to produce the desired bottle speed according to the control algorithm of panel **(A)**. **(C)** Mean and s.e.m. firing rate of a conditioned neuron during all trials of one session as a function of bottle position. The dotted line indicates spontaneous activity calculated during the waiting periods. **(D)** Same as **(C)**, with reconstructed trajectories using the activity of the conditioned neuron during the waiting period, shuffled by blocks of 2 s. One thousand reconstructions were necessary to obtain sufficient coverage of the whole position range. **(E)** Same curves as in **(C)** normalized between 0 and 1, for all the best sessions (one per neuron). The bottle picture shows the starting position. Bins for which the activity values are biased due to the presence of position boundaries have been removed (see Methods). **(F)** Correlation coefficients of the mean activity vs. bottle position, for all conditioned neurons. The right and left halves of the curves in **(E)** were analyzed separately.

In order to gain more insight into how the bottle trajectories are indeed generated by the activity of the conditioned neuron, we looked at how the firing rate depended on the current bottle position and compared our measurements to a prediction elaborated from the neuronal control algorithm. Our operant conditioning protocol is based on the imposed dependence of the bottle speed on the current firing rate of the neuron (Figure 4A). Thus, it does not linearly link the bottle position to the neuronal firing rate. However, it predicts that the neuronal control algorithm should result in a monotonic relationship between these two variables. Indeed, when the bottle is on the right of the drinking zone, the rule calls for an increase in firing rate, resulting in a speed increment toward the left, the larger the further away from the drinking zone (Figure 4B, top right). Conversely, if the bottle is on the left, the firing rate should decrease below the low threshold so that the speed vector again points to the rat's mouth (Figure 4B, bottom left). Thus, given the rule of control of the bottle speed, this intuitive model predicts a monotonic relationship between bottle position and the average activity of the conditioned neuron, with firing rate values for farther positions farther apart from thresholds, so that the bottle travels faster to the drinking zone (Figure 4B). In the drinking zone, we also expect the monotonic curve to hold even though the algorithm produces a null speed in this zone. Indeed, activity values inside the drinking zone that are not between the low and high thresholds should preferably make the bottle move in the correct direction, so that the mean activity for bins on either side of the center can be expected to reflect this bias.

We tested this hypothesis by plotting the mean and standard error of the mean of the neuronal activity of the conditioned neurons at each distance from the mouth, grouped by bins (Figure 4C). As predicted, we observed a monotonic trend with bottle positions. The bin including the starting position of the bottle was an exception due to the presence of boundaries confining the bottle course (see Methods). In order to quantify the observed relationship, we computed activity vs. position for a set of trajectories reconstructed with the waiting period activity, shuffled by blocks of 2 s (see Methods). Activity barely depended on position for this control dataset, except again for the starting position bin. This confirms that the offset found for the starting bin can be attributed to the presence of a boundary. We excluded the bins affected by this bias in the rest of the analysis. The curves for all best sessions, one per neuron, are drawn together in Figure 4E. On the side from which the bottle started, and which required an increase in firing rate for the bottle to move away, all curves displayed an overall profile with a positive slope, matching our prediction. On the opposite side however, the observed curve was sometimes compatible with our prediction, and sometimes flat or with a slope opposite to our prediction. In order to test whether the rat effectively modulates the activity of the cell depending on whether the bottle is to the left or to the right of the target, we compared the firing rate on both sides over equivalent position windows, corresponding to the second and third position bins from the center. The tests for the two bins showed a statistically significant effect of side on firing rate (second position bin from center, *P* < 0.016; third position bin from center, *P* < 0.032, *n* = 7; Wilcoxon paired test). Thus, the rat controls the cell activity differentially on the right and on the left of the drinking zone. In order to quantify more precisely the control in each direction, we fitted two linear functions to each observed curve, one on each side of the rat's mouth. The regression coefficient r was plotted for each side (Figure 4F). For the bottle start side, 6 out of 7 neurons had a correlation coefficient greater than 0.89 and a linear regression *P*-value less than 0.05, confirming the monotonically increasing relation between mean activity and position. For the opposite side, we could distinguish two groups, one with the predicted increasing trend (positive *r*-values), of which only one linear regression was statistically significant (*P* < 0.05), and the other with a decreasing trend (negative *r*-values). One neuron with a limited exploration range on that side showed no clear relationship (green curve). These results suggest that after operant conditioning, the firing rate of a neuron can encode a one-dimensional continuous variable, but that this coding scheme does not always hold for low activity values.

### Bidirectional control with two possible starting positions

**Figure 5:**
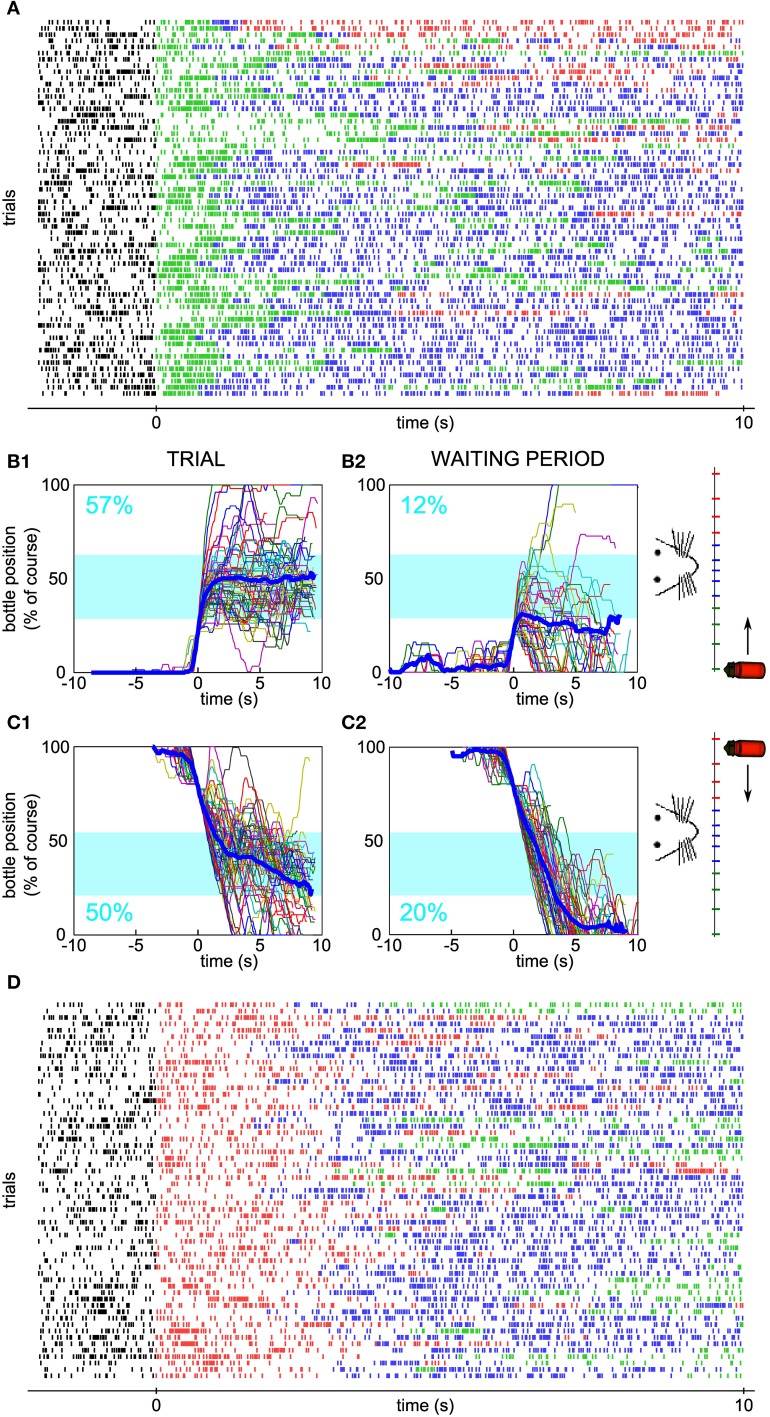
**Neuronal bidirectional control for sessions with different starting positions of the bottle. (A)** Raster plot of the activity of a conditioned neuron, different than the neuron of Figure 2, during a session for which the bottle started from the right and an increase in firing rate was needed at trial onset. Ticks are colored depending on the bottle position as in Figure 1. **(B1)** Superposition of all reconstructed trajectories for the session in **(A)**. The trajectories are centered in time on the position *y* = 20% for clarity, if the bottle reached that position (97% of the trials matched that condition). The percentage of total trial time spent in the drinking zone is displayed in blue. **(B2)** Same as **(B1)**, but using the waiting period activity as an input to the neuronal control algorithm (66% of the trials matched the *y* = 20% condition). **(C)** Similar to **(B)**, but with trajectories for which the bottle started from the left and a decrease in firing rate was needed at trial onset (**C1**: 100% of trials plotted; **C2**: 98% of trials plotted reaching the *y* = 80% condition). **(D)** Raster plot corresponding to the session in panel **(C)**.

In order to confirm that the neuronal output was not a stereotyped reaction to trial onset, we introduced the new requirement of initially decreasing the neuronal firing rate after trial onset in order to move the bottle. Concomitantly, the starting position of the bottle was changed to the other side, so that the same firing-rate-to-speed relationship was enforced (Figure 4A). The four neurons tested in these conditions had all been submitted first to sessions requiring an increase of firing rate (as in Figures 2–3), before being tested in sessions requiring a decrease. In Figure 5, the activity of one neuron is depicted for one session of each type. As previously, we looked at the bottle trajectories during the real trials and compared them to simulated control trajectories. The neuron was significantly conditioned to control the bottle from each side (Initial firing rate increase, Figures 5A,B, z-score = 8.1; Initial firing rate decrease, Figures 5C,D, z-score = 9.3). We observed such a significant conditioning effect for each of the four neurons tested with the requirement of an initial decrease in firing rate (best z-scores for the three additional neurons: 4.1, 4.6, 3.8).

**Figure 6:**
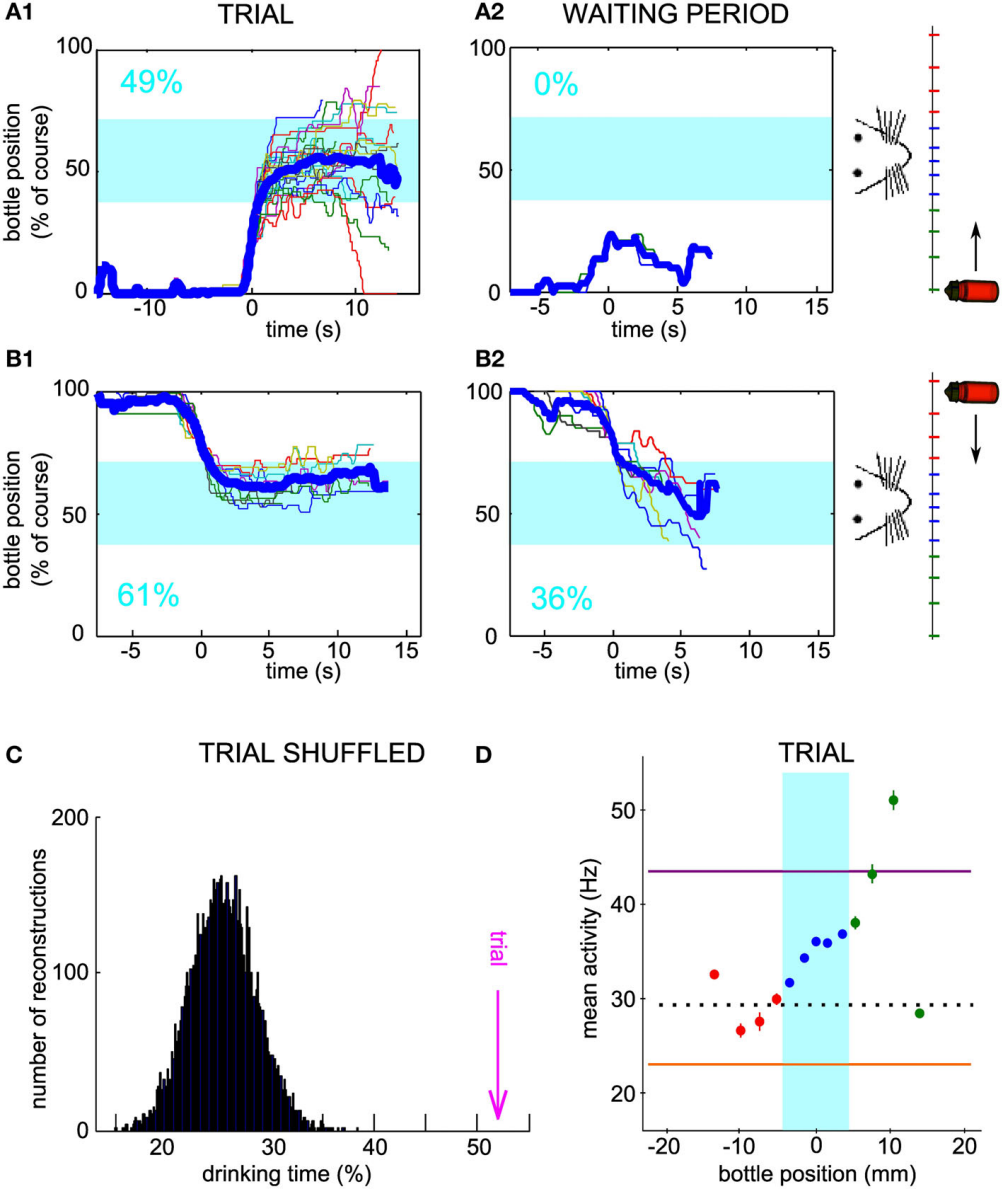
**Neuronal bidirectional control with two possible starting positions of the bottle during the same session. (A1)** Superposition of all reconstructed trajectories for which the bottle started from the right and an increase in firing rate was needed at trial onset, for a conditioned neuron, different than the neurons of Figures 2, 5, trained with two possible starting positions during one session. The trajectories are centered in time on the position *y* = 20% for clarity, if the bottle reached that position (100% of the trials matched that condition). The percentage of total trial time spent in the drinking zone is displayed in the lower left corner in blue. **(A2)** Same as **(A1)**, but using the waiting period activity as an input to the neuronal control algorithm (89% of the trials matched the *y* = 20% condition). **(B)** Similar to **(A)**, but with trajectories for which the bottle started from the left and a decrease in firing rate was needed at trial onset (**B1**: 83% of trials plotted; **B2**: 9% of trials plotted reaching the *y* = 80% condition). **(C)** Distribution of the drinking time percentages obtained with reconstructions using the trial activity shuffled by blocks of 2 s. The bootstrap analysis includes 10,000 shuffles. The value found with real trial activity is indicated by the vertical magenta arrow. All drinking time values are averages taking into account both the left and right starting positions datasets. **(D)** Mean and s.e.m. firing rate of the conditioned neuron as a function of bottle position during the same session. Horizontal lines indicate the high threshold (purple), the low threshold (orange) and the average activity during the waiting period (dotted line). All trials are included, whether starting from the left or right positions.

Furthermore, we were able to test, for one conditioned neuron only, whether the operant conditioning could be achieved when the starting position of the bottle was alternated between left and right, by blocks, inside one session. Indeed, for both starting positions, the bottle followed trajectories entering and staying in the drinking zone (Figures 6A1–B1) more often than expected by chance, as assessed by using the waiting period activity for the simulated trajectories (Figures 6A2–B2). Again, we verified that a single firing rate change could not account for the neuronal control during trials. We calculated the drinking time distribution obtained from shuffled trial activity and confirmed that the real drinking time was well above the distribution (Figure 6C, z-score = 8.6; Right-to-Left trials only, z-score = 4.6; Left-to-Right trials only, z-score = 7.3). The mean activity showed a monotonically increasing profile with bottle position, as predicted for a successful control. Overall, these results strengthen the idea that single neurons can indeed adapt their firing rate in real time to a one-dimensional variable, rather than repeat a unique pattern of activity from trial to trial.

### Faster reaction and better control of the conditioned neuron

**Figure 7:**
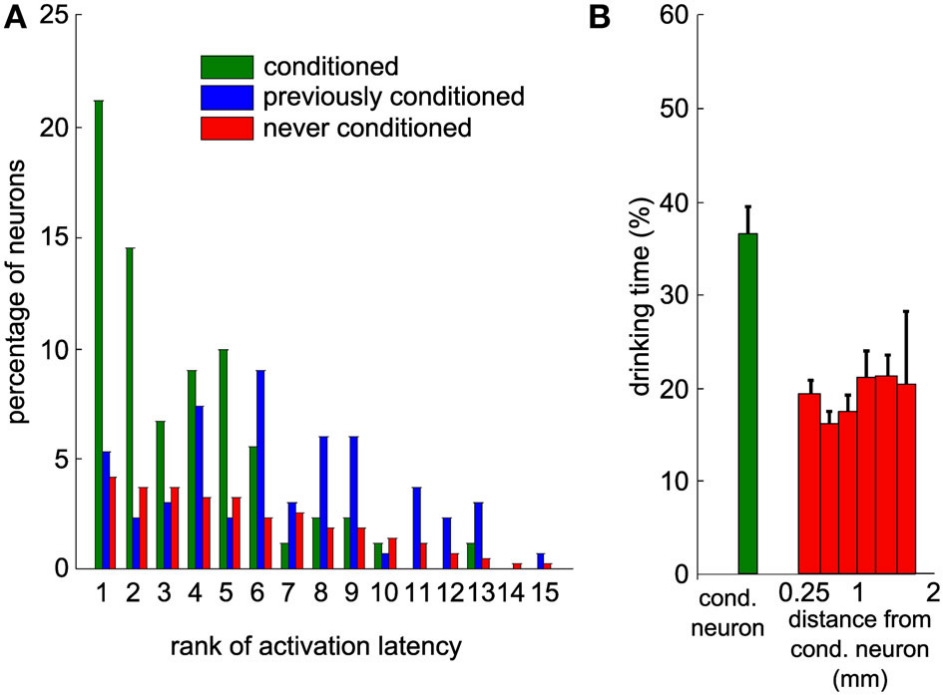
**Neighboring neurons recorded simultaneously display delayed modulations of activity that would have led to a lower task performance. (A)** Distribution of the ranks of activation for all sessions and all neurons, based on the latencies of increase in firing rate above baseline. This includes only the sessions where this latency could be measured for at least one neuron. All the neurons are partitioned into three categories (green: conditioned during that session; blue: previously conditioned; red: never conditioned; overall, 1993 values from 155 neurons and 88 sessions). **(B)** Percentage of time during which the bottle was in the drinking zone, using the activity of each of the recorded neurons during trials as an input to the control algorithm to simulate bottle trajectories. The first bin contains all conditioned neurons, while other bins contain all other neurons grouped by distance to the conditioned neuron. Thresholds on the firing rate histograms for the control algorithm were set to the same percentiles than during the real experiment for the conditioned neuron. Vertical bars represent s.e.m.

Given the high level of control of the actuator by the neuronal activity of the conditioned unit, we studied the activity changes of the local neuronal network around the conditioned neuron. We analyzed the activity of neighboring neurons simultaneously recorded but that were not used by the conditioning algorithm. Many neurons showed an increase in activity after trial onset. We quantified the baseline activity of each neuron in the waiting period by its mean value and standard deviation (SD). The reaction time of each neuron was defined as the time after trial onset for which the activity first exceeded the mean firing rate + 2 SD (see Methods). Based on these latencies, we ranked all neurons recorded simultaneously and observed that the conditioned neuron increased its firing rate on average before non-conditioned surrounding neurons (Figure 7A, Mann-Whitney *U*-test between the conditioned and never-conditioned rank distributions, *P* < 8.10^−23^). Interestingly, the conditioned neuron was faster also than neurons that had been conditioned in previous sessions (Mann-Whitney *U*-test, *P* < 5.10^−8^). This suggests that when the experimenter changes the reference neuron on which the control algorithm is applied, the order of activation in the network is updated in subsequent sessions to reflect that change.

To further support the idea that the conditioned neuron reacted more specifically than the rest of the population, we simulated bottle trajectories by driving the control algorithm with the activity of each recorded neuron, with thresholds set at the same percentiles as for the conditioned neuron. Each of these simulated sessions gave us a percentage of trial time spent in the drinking zone. Figure 7B displays a histogram of the drinking time as a function of the distance between recording sites of the non-conditioned neuron used for trajectory reconstruction and of the conditioned neuron. The real drinking time, displayed for the conditioned neuron, was significantly higher than the drinking time simulated with activity from non-conditioned neurons (Mann-Whitney *U*-test, *P* < 4.10^−5^). No spatial trend could be observed for the non-conditioned neurons. This indicates that motor cortex neurons in the local network do not systematically modulate their activity as strongly and as efficiently as the neuron that was conditioned in our protocol.

## Discussion

We recorded activity from 155 neurons in the freely behaving rat motor cortex and applied operant conditioning on seven units, using the instantaneous firing rate of those neurons to control in real-time a bottle placed on a one dimensional actuator. We demonstrate, during behavioral learning, the adaptation of the neuronal firing rate of the conditioned neuron to the instantaneous position of the bottle during trials of 10 s. This firing rate modulation occurred while the head-fixed rat drank at the same time. A better task performance, quantified by the duration of reward obtention, was established from the activity of the conditioned neurons than that predicted from the spiking patterns of non-conditioned neurons recorded simultaneously.

In the current study, we focused on the late stages of learning of an operant conditioning task to evaluate how precisely motor neurons could control an actuator moving in both directions on a linear axis, by modulating up or down their firing rate. This followed an initial training phase where the neurons only had to increase their firing rate to move the bottle in a single direction. In the bidirectional task, the firing rate needed to be brought in one of three zones defined by two thresholds, depending on whether the bottle was currently on the right, on the left, or correctly placed in the drinking zone in front of the rat's mouth. Our results confirm that the neurons did adjust their firing rate during trial compared to the waiting period within trials, optimizing the time spent in the drinking zone and thus reward obtention. Indeed, during most sessions, the trajectories of the bottle spent on average more time in the drinking zone than trajectories reconstructed with the waiting period activity, or with shuffled trial activity. The differences between those integrated drinking times were largely significant for the best sessions of the seven neurons conditioned.

Other studies have trained neurons to generate different firing rates depending on the targets to match, using the activity of a few units at the same time in the rat (Gage et al., 2005; Marzullo et al., 2006; Koralek et al., 2012) or using only one neuron in the monkey (Moritz et al., 2008). This latter experiment showed that conditioning was possible in only one session, and for all neurons tested. By contrast, in our rat study, significant operant conditioning required a long sequence of training sessions, first in a simple unidirectional version of the task, and then in the bidirectional version. Four factors could explain that difference. First, the rat behavior was possibly altered by the head fixation, which could slow down learning. Second, the reward time was not separated from the task trials, that is, the rat had to control the bottle while trying to get the liquid reinforcement. This temporal contingency of operant behavior and reward could be an additional source of complexity. Third, as the controlled parameter was the bottle speed, and not directly the bottle position, one difficulty of the task was that the target firing pattern that had to be matched constantly changed depending on the instantaneous bottle position relative to the rat mouth. This is in contrast to other experiments, where each target corresponded to a fixed predefined firing pattern. This difficulty of our protocol could have required additional preparation or trial processing time, or even the establishment of an internal model of the actuator. Last, the neurons used in the experiment by Moritz and collaborators were found in the wrist region of the macaque motor cortex, an area from which monosynaptic connections to muscle motoneurons exist, useful for fine control of distal movements and for the development of adaptive motor programs. Such monosynaptic connections are absent from the rat motor cortex (Lemon, 2008). The fact that the output of a conditioned neuron has a less direct link to body movements in the rat than in the monkey could mean that more complex mechanisms are underplay for inducing plasticity changes on the conditioned neuron and its local network.

We examined how the firing rate of the conditioned neuron was effectively modulated in order to produce the observed task performance. We computed the mean firing rate along the length of the bottle course. We expected to find a monotonic curve, that is, with points farther away from the center leading to larger speeds in the appropriate direction, so that the bottle would converge to the rat's mouth. This profile was observed at least partially on all neurons. More precisely, we found that on the side requiring an increase in firing rate for appropriate bottle movement, the curve indeed showed a clear increasing slope for all neurons. On the side requiring a decrease in firing however, we observed no systematic relationship. This asymmetry could be explained by three different mechanisms. First, some of the selected neurons had a rather low spontaneous activity and it is possible that they could intrinsically increase their firing rate more easily than decrease it. When the bottle was in the drinking zone, the rat could readily drink and neuronal activity remained low. When the bottle was outside the drinking zone, in either direction, the rat could have been trying any change in behavior in order to get the reward, the attempted behavior causing most often an increase in firing rate in the conditioned neuron rather than a decrease, and therefore a bimodal curve. Second, the unidirectional initial training was based on an increase in firing rate only (Arduin et al., 2013). Therefore, some neurons might have developed a higher capability for that modulation due to a priming effect, and more time would have been needed to learn the opposite modulation. Third, except for a minority of sessions, the bottle always started on the side requiring an initial increase in firing rate, and a trial could actually be completed only by exceeding the high activity threshold for an appropriate amount of time and staying inside the thresholds thereafter. This asymmetry in the activity requirement could well have led to a stronger association between increases in firing rate with the reward rather than decreases, and could have impeded the learning on the side of the mouth opposite to the starting position. The first reason would be problematic if transposing the protocol to the human, whereas the two others, related to the conditioning history of the neuron, could reasonably be overcome by a different training protocol. In any case, some of the neurons managed to properly control the bottle speed in each direction, as shown by trajectories that left the drinking zone but reliably re-entered it. We also observed successful conditioning for four neurons for which the bottle starting position was changed to the other side, now requiring an initial decrease in firing rate (Figure 5). Furthermore, one neuron controlled the bottle from both initial sides during the same session, with trials alternating in blocks between right and left starting positions (Figure 6). The difficulty in testing neurons for a large number of sessions, as seems necessary for mastering this final two-side version of our protocol, prevented us from collecting more data. Future experiments will be needed to confirm that a full two-sides bidirectional operant conditioning can be achieved using a single neuron of the motor cortex. Nonetheless, our results already support the observation that the neuronal modulation of activity was more than a mere systematic reaction after trial onset.

Finally, the ultimate objective of such protocols is to assign several small groups of neurons to different parts of a robotic device. For individuated finger control for example, dissociation of the output of those groups is required, as synergies between fingers do not seem to dominate (Valero-Cuevas et al., 2009). In a previous study, we discovered that the neurons in the part of the motor cortex we recorded from reacted less strongly and less quickly than the conditioned neuron, when a unidirectional control algorithm was implemented (Arduin et al., 2013). Here, we confirm that non-conditioned neighboring neurons exhibited delayed activity changes that would have led to a smaller drinking time on average. These results indicate that the conditioning of the motor cortex was not uniform, and bode well for the conditioning of distinct groups of neurons, as was previously carried out for motoneurons (Smith et al., 1974), motor units (Basmajian, 1963), and neurons and muscles (Fetz and Finocchio, 1971; Fetz and Baker, 1973; Moritz et al., 2008).

In conclusion of this study, we report that single neurons in the rat motor cortex were suitable to control a one-dimensional actuator in real-time, after operant conditioning. The control was maintained for several seconds, with firing rate changes depending on the instantaneous bottle position. The modulation of activity was maintained while the rat licked the tip of the bottle when close enough to its mouth. These results suggest that brain-machine interfaces based on the control of actuators by single units should be further tested, along with the brain-machine interfaces based on decoding paradigms.

### Conflict of interest statement

The authors declare that the research was conducted in the absence of any commercial or financial relationships that could be construed as a potential conflict of interest.
