# Supplementary figures and images for: Bidirectional control of a one-dimensional robotic actuator by operant conditioning of a single unit in rat motor cortex
Source: Front Neurosci. 2014 Jul 25;8:206. doi: 10.3389/fnins.2014.00206 (PMC4110947; doi:10.3389/fnins.2014.00206)

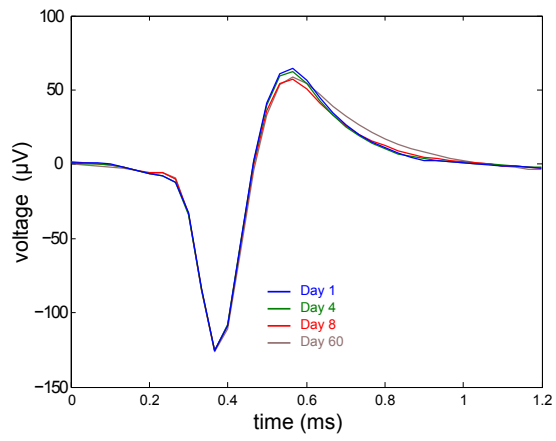

Supplementary Figure. Average waveform of a conditioned neuron across 60 days

Supplement: Supplementary file 1 [file Presentation1.PDF]
